# α-((4-Cyanobenzoyl)oxy)-ω-methyl poly(ethylene glycol): a new stabilizer for silver nanoparticles

**DOI:** 10.3762/bjnano.8.67

**Published:** 2017-03-15

**Authors:** Jana Lutze, Miguel A Bañares, Marcos Pita, Andrea Haase, Andreas Luch, Andreas Taubert

**Affiliations:** 1 German Federal Institute for Risk Assessment (BfR), Department of Chemical and Product Safety, Max-Dohrn-Strasse 8-10, D-10589 Berlin, Germany; 2 Instituto de Catálisis y Petroleoquímica, ICP-CSIC, C/ Marie Curie 2, E-29049 Madrid, Spain; 3 Institute of Chemistry, University of Potsdam, Karl-Liebknecht-Str. 24-25, D-14476 Golm, Germany

**Keywords:** cyano anchor group, poly(ethylene glycol), polymer coating, silver nanoparticles

## Abstract

The article describes the synthesis and properties of α-((4-cyanobenzoyl)oxy)-ω-methyl poly(ethylene glycol), the first poly(ethylene glycol) stabilizer for metal nanoparticles that is based on a cyano rather than a thiol or thiolate anchor group. The silver particles used to evaluate the effectiveness of the new stabilizer typically have a bimodal size distribution with hydrodynamic diameters of ca. 13 and ca. 79 nm. Polymer stability was evaluated as a function of the pH value both for the free stabilizer and for the polymers bound to the surface of the silver nanoparticles using ^1^H NMR spectroscopy and zeta potential measurements. The polymer shows a high stability between pH 3 and 9. At pH 12 and higher the polymer coating is degraded over time suggesting that α-((4-cyanobenzoyl)oxy)-ω-methyl poly(ethylene glycol) is a good stabilizer for metal nanoparticles in aqueous media unless very high pH conditions are present in the system. The study thus demonstrates that cyano groups can be viable alternatives to the more conventional thiol/thiolate anchors.

## Introduction

Nanoparticles (NPs), especially metal NPs such as gold and silver NPs (SNPs), are currently among the most popular research subjects and many particles have been suggested for applications in, e.g., electronics or medicine [1–3]. Metallic nanoparticles intended for use in medicine or diagnostics are generally modified with an organic coating designed to prevent or at least to reduce aggregation in aqueous media [4–13]. A good stabilizing agent is thus essential to obtain and retain stable particle dispersions [11,14–16].

Synthetic particles often have coatings made from polymers such as poly(vinyl pyrrolidone), poly(vinyl alcohol), or poly(ethylene glycol) (PEG). PEG-based coatings are particularly popular [17–18]. This is also due to the high hydrophilicity and biological inertness of PEG, which renders PEG-stabilized NPs water-soluble and less susceptible to opsonization [19–20]. As a result, PEG-coated NPs are often referred to as “stealth” NPs [21–22]. Moreover, PEG coatings often lower the toxicity of otherwise toxic NPs [23–24]. For example, Zhang et al. [25] demonstrated the passivating effect of a PEG/silica hybrid coating on CdSe quantum dots. Thus, there is a growing interest in PEG-based stabilizers for a large variety of applications including catalysis, diagnostics, biology or biomedicine.

There are numerous examples in which the oligomeric or polymeric stabilizer is attached to the particle surface without a specific anchor group [26–30]. The current gold standard for anchoring an organic moiety on the NP surface is the thiol or thiolate group [3,24,31–33]. There are, however, cases where the thiol group may not be desirable (such as situations, where thiols may form thiyl radicals leading to protein degradation and cause diseases such as cancer) [34–35]. Hence, there is an interest in alternative anchoring groups with a similar or better anchoring efficiency. Among the possible candidates are the amine and the cyano groups. Especially the cyano group is interesting because of its relatively high metal binding capability (although lower than that of the thiol group) [36] and its stability in a variety of chemical environments [37]. Yet, the number of reports on stabilizers for metal nanoparticles with a cyano group anchor is limited [36,38–43] and further development of cyano-based stabilizers is of high interest. However, depending on the application, the toxicity of the cyano-based stabilizers will also have to be considered [44].

The current study introduces a new stabilizer, α-((4-cyanobenzoyl)oxy)-ω-methyl poly(ethylene glycol) (CBAmPEG) exploiting the cyano group as an anchor. CBAmPEG is (i) accessible in good yield via a simple one-step synthesis and (ii) provides an efficient stabilization of SNPs in a pH range between 3 and 9. The latter point is important because degradation of the stabilizer may lead to particle aggregation and sedimentation, which is often –for example in a biomedical context [10–11,45] – highly undesirable and must be avoided. The new CBAmPEG introduced here is thus an attractive alternative to other polymeric stabilizers and to the best knowledge of the authors it is the first PEG-based stabilizer for metal nanoparticles with a cyano anchor group.

## Experimental

### General

4-Cyanobenzoic acid (98%, abcr), 4-(dimethylamino)pyridine (99%, Aldrich), deuterium oxide (99.9 atom % D, Aldrich), hydrochloric acid (37%, lab reagent grade, Fisher Scientific), L-ascorbic acid (≥99%, Roth), *N*,*N’*-dicyclohexylcarbodiimide (99%, Aldrich), poly(ethylene glycol) methyl ether (*M*_n_ = 4830 g·mol^−1^, Aldrich), silver nitrate (≥99%, Sigma-Aldrich), sodium hydroxide (>99%, Roth), and trisodium citrate dihydrate (99.7%, Merck) were used as received. Water with a resistivity of 18.2 MΩ·cm (Milli-Q) was used for particle synthesis and purification.

### Synthesis of CBAmPEG

4-Cyanobenzoic acid (0.6 g, 4 mmol), poly(ethylene glycol) methyl ether (5.0 g, 1 mmol, *M*_n_ = 4830 g·mol^−1^) and 4-(dimethylamino)pyridine (DMAP, 50 mg, 0.4 mmol) were dissolved in dichloromethane (DCM, 10 mL). The solution was cooled to 0 °C and *N*,*N’*-dicyclohexylcarbodiimide (DCC, 1.0 g, 5 mmol) in dichloromethane (1 mL) was added slowly. After ten minutes of stirring the reaction mixture was warmed to room temperature and stirred overnight. The precipitate was filtered off and the filtrate was precipitated in *tert*-butyl methyl ether. The product was dissolved in distilled water and insoluble parts were removed by filtration. Lyophilization afforded the pure product as a colorless powder. Yield: 3.8 g (74%). The number of repeating units of CBAmPEG was calculated from the molar mass derived from gel permeation chromatography (GPC) measurements with refractive index (RI) detector.

^1^H NMR (300 MHz, D_2_O) δ 8.05 (m, 4H), 4.53 (m, 2H), 3.67 (m, 488H); ATR-IR (cm^−1^): 2947 (sh, v(CH)), 2885 (s, v(CH)), 2742 (vw, v(CH)), 2696 (vw, v(CH)), 1724 (w, v(C=O)), 1466 (m, δ(CH_2_)), 1412 (w, δ(CH_2_)), 1358 (w, δ(CH_2_), v(CC)), 1342 (m, δ(CH_2_)), 1281 (m, δ(CH_2_)), 1242 (m, δ(CH_2_)), 1146 (m, v(C-O)), 1107 (s, v(C-O)), 1061 (m, δ(CH_2_), v(C-O)), 960 (m, δ(CH_2_)), 845 (m, δ(CH_2_)), 768 (vw, δ(C=O)), 694 (vw, δ(Ph)), 528 (vw, δ(OCC)); Raman (cm^−1^): 2953 (sh, v(CH)), 2934 (s, v(CH)), 2897 (sh, v(CH)), 2237 (m, v(CN)), 1612 (m, v(Ph)), 1480 (m, δ(CH_2_)), 1449 (w, δ(CH_2_)), 1306 (m, δ(CH_2_)), 1287 (m, δ(CH_2_)), 1132 (m, v(C-O)), 1054 (m, v(C-O), v(CC), v(CH_2_)), 854 (m, v(C-O), v(CH_2_)); GPC: *M*_n_(UV) = 4627 g·mol^−1^; PDI(UV) = 1.0544; *M*_n_(RI) = 4578 g·mol^−1^, PDI(RI) = 1.0654; Anal. calcd: C, 54.9; H, 9.0; found: C, 54,8; H, 8.3.

### Silver nanoparticle synthesis

**Scheme 1:**
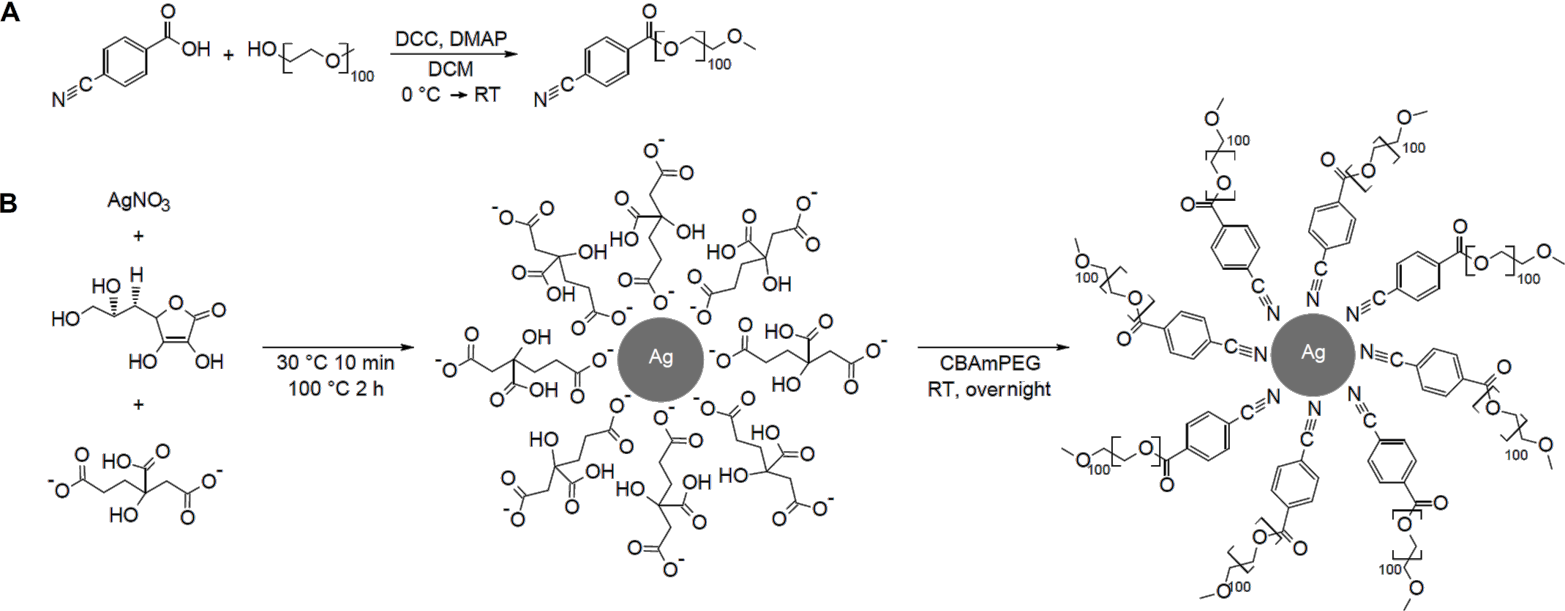
Synthesis of A) α-((4-cyanobenzoyl)oxy)-ω-methyl poly(ethylene glycol) (CBAmPEG) via Steglich esterification and B) synthesis of CBAmPEG-coated SNPs (CBAmPEG@SNPs). Note that the exchange between citrate and CBAmPEG is not complete (see details below) and the final product is a mixed coating.

Citrate-coated SNPs were synthesized according to a procedure of Qin et al. [46] 4 mL of a 1.2·10^−3^ M aqueous ascorbic acid solution (4.8 µmol) and 4 mL of a 6·10^−3^ M aqueous trisodium citrate dihydrate solution (24 µmol) were combined and the pH was adjusted to 10.5 with a 0.5 M sodium hydroxide solution. The mixture was heated to 30 °C and a silver nitrate solution (0.1 M, 80 µL, 8.0 µmol) was added under vigorous stirring. After ten minutes stirring at room temperature the mixture was refluxed for two hours, resulting in an orange dispersion, which was allowed to cool to room temperature before adding CBAmPEG (200 mg, 44 µmol) for the ligand exchange reaction. After stirring the mixture overnight at room temperature under the exclusion of light, the dispersion was centrifuged three times at 8000 rpm for 60 min. The sediment was redispersed in DI water or purified by dialysis against water for 24 h (MWCO 100 kDa). Scheme 1 summarizes polymer and nanoparticle synthesis.

Raman (cm^−1^): 2930 (s, v(CH)), 2247 and 2237 (m, v(CN)), 1648 (w, v(C=C)), 1605(w, v(Ph)), 1573(m, v(CO_2_), 1508 (w, δ(CH_2_)), 1443 (w, δ(CH_2_)), 1390 (sh, δ(CH_2_)), 1362 (m, v(CO_2_)), 1310 (w, δ(CH_2_)), 1285 (sh, δ(CH_2_)), 1234 (w, δ(CH_2_)), 1170 (s, v(C-O)), 1128 (vw, v(CC), δ(CH_2_)), 1032 (w, v(C-O), v(CC), δ(CH_2_)), 1002 (w, v(C-O), v(CC), δ(CH_2_)), 944 (w, v(C-O), v(CC), δ(CH_2_)), 889 (vw, v(C-O), δ(CH_2_)), 819 (w, v(C-O), δ(CH_2_)), 773 (w, 1,4-disubstituted aromatic ring bending), 715 (m, δ(CO)), 675 (m, δ(CO)), 659 (sh, δ(CO)), 613 (m, scissoring NO_2_), 569 (vw, CN in plane bending), 523 (w, δ(OCC)), 504 (sh, 1,4-disubstituted aromatic ring bending), 396 (vw, δ(CCN), 347 (vw, δ(COC), δ(OCC)), 311 (vw, possibly due to general skeletal vibrations).

### Cleavage experiments

For evaluating the cleavage of the pure CBAmPEG polymer, D_2_O solutions with a pH value of 3, 6, 9, and 12 were prepared by dissolving an appropriate amount of sodium hydroxide or concentrated hydrochloric acid in D_2_O and adjusting the pH with 0.1 M sodium hydroxide or 0.1 M hydrochloric acid. The differences of pH, pD, and pHD values were not considered. CBAmPEG was then dissolved in each of the pH-adjusted D_2_O solutions at a concentration of 0.02 mol/L and the mixtures were heated to 37 °C. After 6, 12, 18, and 24 h ^1^H NMR spectra of each solution were recorded.

Evaluation of the cleavage of the polymer on the silver nanoparticles (CBAmPEG@SNPs) was done as follows: the pH of a dialyzed dispersion of CBAmPEG@SNPs (2.5 mL) was adjusted to 12. This sample and a control sample (dialyzed dispersion without pH adjustment, pH ca. 7) were incubated at 37 °C in the dark for 66 h without stirring. The experiment was repeated three times with three different SNP batches. For the control experiments a change in color from yellow to orange was observed in two of three cases, whereas for the samples with an adjusted pH of 12 a small amount of solid floating at the surface of the reaction vessels could be observed. Samples were removed from the heating bath for DLS and zeta-potential measurements.

### Analytical methods

**Gel permeation chromatography.** GPC measurements were done on two 300 × 8 mm^2^ PSS-GRAM (7 µm particles) columns with porosities of 10^2^–10^3^ Å in *N*-methyl-2-pyrrolidone (NMP + 0.5 wt % LiBr at 70 °C and a flow rate of 0.8 mL/min with simultaneous UV and RI detection. Polyethylene oxide (PEO) standards (PSS, Mainz, Germany) were used for calibration.

**Transmission electron microscopy.** Samples were centrifuged three times to remove residual polymer not attached to the NP surface. For TEM measurement the samples were diluted and dispersed in an ultrasonic bath for 15 min. Afterwards a drop of the diluted and ultrasound-treated sample was deposited on a carbon film coated 200 mesh copper TEM grid (Electron Microscopy Sciences) and allowed to dry. A 200 kV JEOL 2100 transmission electron microscope equipped with an Oxford Instruments EDX analyzer was used for the analysis.

**IR spectroscopy.** IR spectra were measured with a Thermo Nicolet Nexus FTIR spectrometer with a Thermo Scientific Smart Orbit (Diamond). ATR correction was done with Omnic 8.1.11 from Thermo Nicolet Fischer Scientific. For IR spectroscopy, a few microliters of SNP dispersion were deposited on the ATR crystal and allowed to dry in air for a few minutes. Spectral resolution was 4 cm^−1^ and spectra were acquired from 400 to 4000 cm^−1^.

**Raman spectroscopy.** Raman spectroscopy measurements were performed on a Renishaw System 1000 Raman Spectrometer with 514.5 nm tuned solid-state excitation laser and an edge filter to remove elastically scattered light, and a Peltier-cooled CCD detector (−70 °C). Raman spectra were acquired with a laser power of 1 mW on sample; 10 spectra of 6 s were acquired for each sample. Samples were used as obtained from particle synthesis, that is, no dilution or other sample processing was performed prior to Raman analysis.

**NMR spectroscopy.** ^1^H NMR spectra were recorded on a Bruker Avance 300 MHz or 500 MHz spectrometer in D_2_O with TMS as an internal standard.

**Light scattering and zeta potential.** The hydrodynamic diameter *R*_h_ and the zeta potential of the SNPs were determined with a Zetasizer ZS and the Zetasizer 7.1 Software (Malvern Instruments, UK). For DLS experiments the samples were diluted with DI water (18.2 MΩ) and placed in disposable plastic cuvettes. For each sample five measurements consisting of ten runs of ten seconds each were done. The laser wavelength was 633 nm and data were recorded in backscattering mode 2θ = 173° at a temperature of 25 °C. Electrophoretic mobilities were measured five times for each sample in an automatic mode (10 to 100 runs per measurement) at 25 °C and automatically converted to zeta potentials using the Smoluchowski model.

## Results

### α-((4-Cyanobenzoyl)oxy)-ω-methyl poly(ethylene glycol) (CBAmPEG)

**Figure 1:**
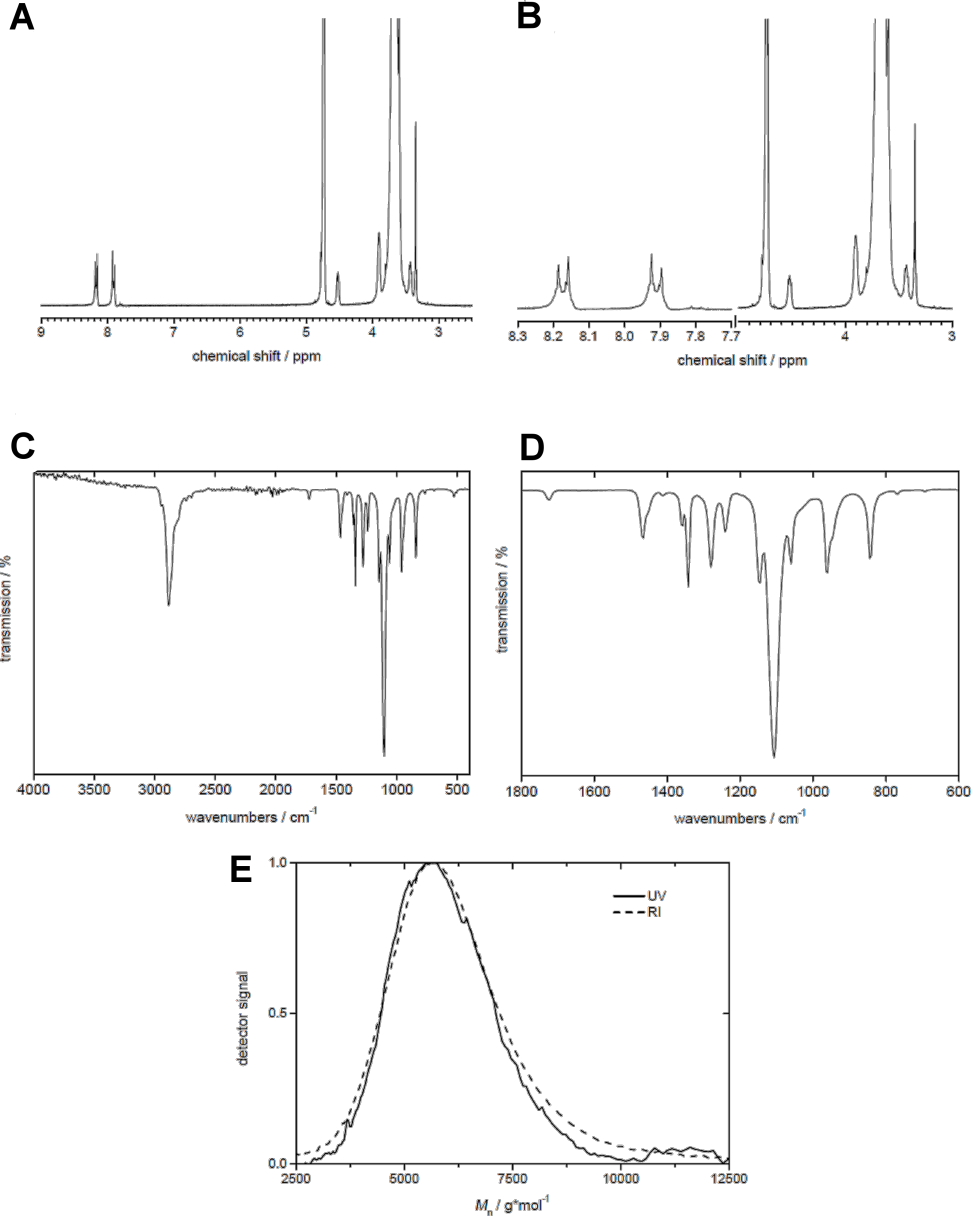
A) ^1^H NMR spectrum, B) magnified view of the NMR signals, C) overview FTIR spectrum, and D) detailed view of the spectral region with the most intense signals, E) GPC data of CBAmPEG. NMR and IR bands are listed in the Experimental section.

CBAmPEG was synthesized via Steglich esterification of 4-cyanobenzoic acid and poly(ethylene glycol) methyl ether (*M*_n_ = 4830 g·mol^−1^) as shown in Scheme 1. The ^1^H NMR and the IR spectra of the product confirm the successful formation of CBAmPEG, Figure 1. The ATR-IR spectra show bands at 2947, 2885, 2742, 2696, 1466, 1412, 1358, 1342, 1281, 1242, 1061, 960, and 845 cm^−1^ that can be assigned to various C–H vibrations (see Experimental section for specific assignments). Bands at 1724, 1146, 1107, and 768 cm^−1^ can be assigned to C=O and C–O vibrations. Finally, a band at 694 cm^−1^ is due to phenyl ring bending vibrations. Signals in the ^1^H NMR spectra are due to the aromatic protons of the phenyl group (8.05 ppm), the methylene (4.53 ppm, 2 H atoms) protons adjacent to the ester linker unit, and the methylene groups of the PEG chain (3.67 ppm, 488 H atoms). The terminal methyl group of the PEG chain is not visible in the NMR spectra. These data are confirmed by elemental analysis (experimental part) and GPC (Figure 1) proves that the product CBAmPEG has a monomodal molecular weight distribution.

### CBAmPEG-modified SNPs (CBAmPEG@SNPs)

Citrate-coated SNPs (citrate@SNP) [46] were reacted with CBAmPEG at room temperature to obtain SNPs coated with CBAmPEG (CBAmPEG@SNP). The particles were characterized with Raman spectroscopy, dynamic light scattering (DLS), and transmission electron microscopy (TEM).

**Figure 2:**
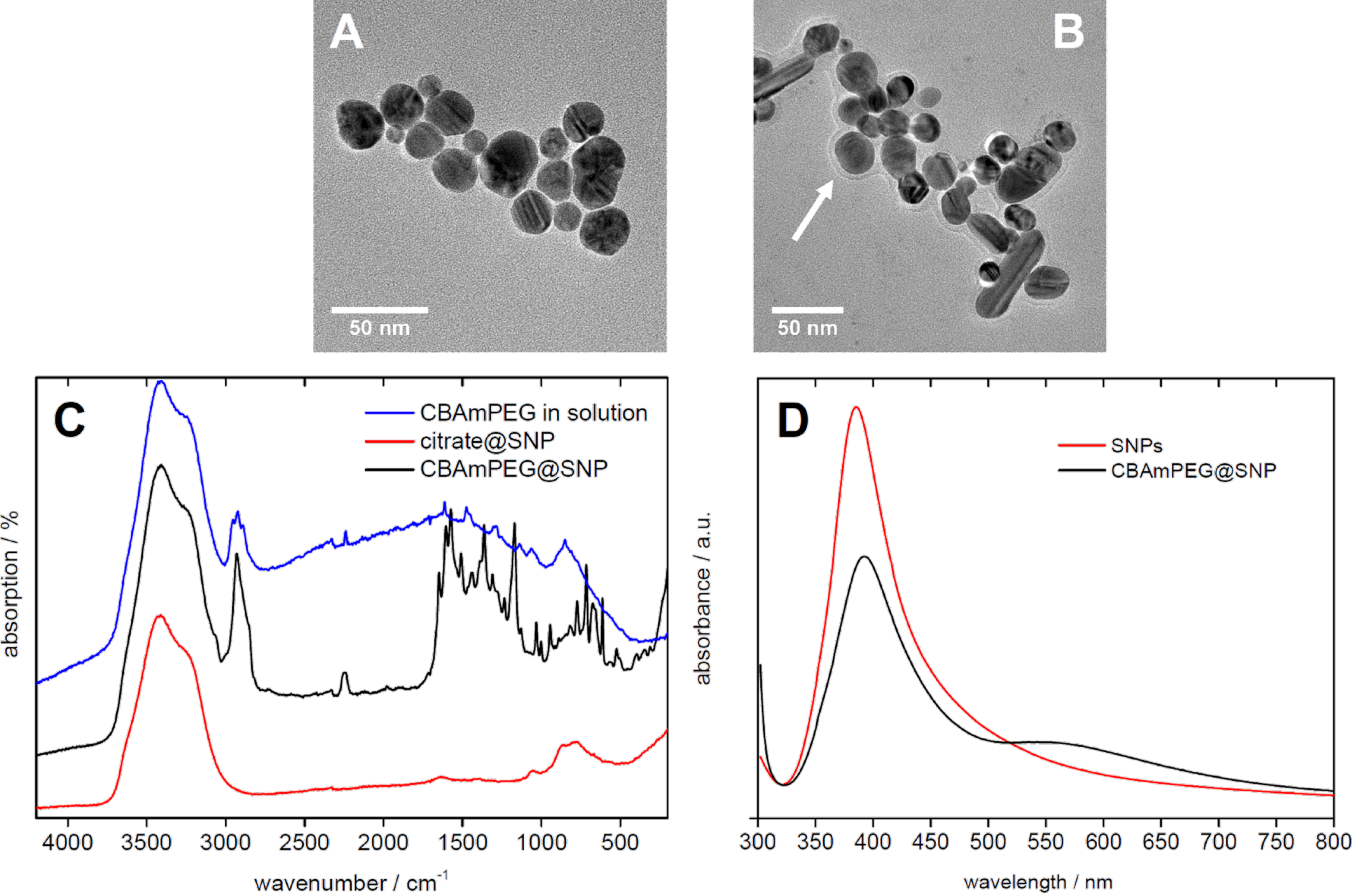
TEM images of A) citrate@SNPs and B) CBAmPEG@SNPs. C) Raman spectra of aqueous solutions or suspensions of CBAmPEG, CBAmPEG@SNPs, and citrate@SNPs. A detailed assignment of the Raman bands is given in the Experimental section. D) UV–vis spectra of SNP without polymer modification and CBAmPEG@SNP.

TEM images of citrate@SNP and CBAmPEG@SNP (Figure 2) show spherical to ellipsoidal particles with diameters in the range of 10 to 40 nm with a broad size distribution. Some samples (Figure 2B) show a fair amount of rod-like particles, typical of the synthesis using ascorbic acid. After modification with CBAmPEG, the resulting CBAmPEG@SNP nanoparticles show a halo around each silver core demonstrating that the particles are indeed modified with the polymer coating. The polymer layer is (in the vacuum-induced dry state in the TEM) about 4 nm thick. Further analysis of CBAmPEG@SNP in aqueous dispersion at pH 7 via dynamic light scattering (DLS) yields a hydrodynamic radius R_h_ of around 60 nm after dialysis; often the samples have a bimodal size distribution (Table 1).

**Table 1:** Zeta potential and *R*_h_ of CBAmPEG@SNPs incubated at 37 °C for 66 h at pH 7 and pH 12 from three independent experiments.

	zeta potential (mV)		*R*_h_ (DLS, nm), peak intensities (%) in brackets			
experiment #	pH 7	pH 12	pH 7	PDI	pH 12	PDI

1	−20(1)	−50(2)	9 ± 3 (6.8%); 68 ± 31 (100%)	0.278	11 ± 3 (4.7%); 68 ± 34 (95.3%)	0.283
2	−21(1)	−42(2)	11 ± 3 (5.9%); 70 ± 29 (94.1%)	0.240	64 ± 28 (100%)	0.244
3	−29(1)	−42(3)	46 ± 14 (100%)	0.221	54 ± 23 (100%)	0.312

Figure 2C shows Raman spectra of a dispersion of CBAmPEG@SNP and of an aqueous solution of the pure polymer CBAmPEG. The intense signal at 3400 cm^−1^ and a weak broad mode near 1600 cm^−1^ derives from the OH-stretching and bending modes of water, respectively. The signal at 2230 cm^−1^ observed in the cases of the pure polymer and CBAmPEG@SNP but not for citrate@SNP can be assigned to the CN-stretching mode of the nitrile group of the polymer. The blue shift in the spectrum obtained from CBAmPEG@SNP must be related to the polymer being attached to the SNP. Moreover, the clear signal enhancement of the polymer bands in the CBAmPEG@SNP samples prove that the polymer is indeed anchored to the SNP surface. Finally a band at 613 cm^−1^ is due to a scissoring NO_2_ vibration, likely from nitrate anions adsorbed on the SNP surface [47]. It must be noted here that at an excitation wavelength of 514.5 nm, the surface enhancement effect is less pronounced than at other wavelengths, but nevertheless is an established approach [48–54].

Figure 2D shows that the UV–vis spectra exhibit significant changes upon treatment of the nanoparticles with the polymer. Initially the samples exhibit a single, rather broad plasmon band at 385 nm. After modification with the polymer, two bands at 393 and 545 nm are visible.

### Stability of CBAmPEG in solution and on the SNP surface

One of the most important parameters that affect polymer and particle stability and behavior is the pH value. We have studied the effects of pH variation on polymer degradation both in solution of the pure polymer and on the SNP surface. The least stable functional group in our model system is the ester group linking the PEG chain to the cyanobenzoic acid group. As a result, a cleavage of the ester group is likely the first degradation step.

**Figure 3:**
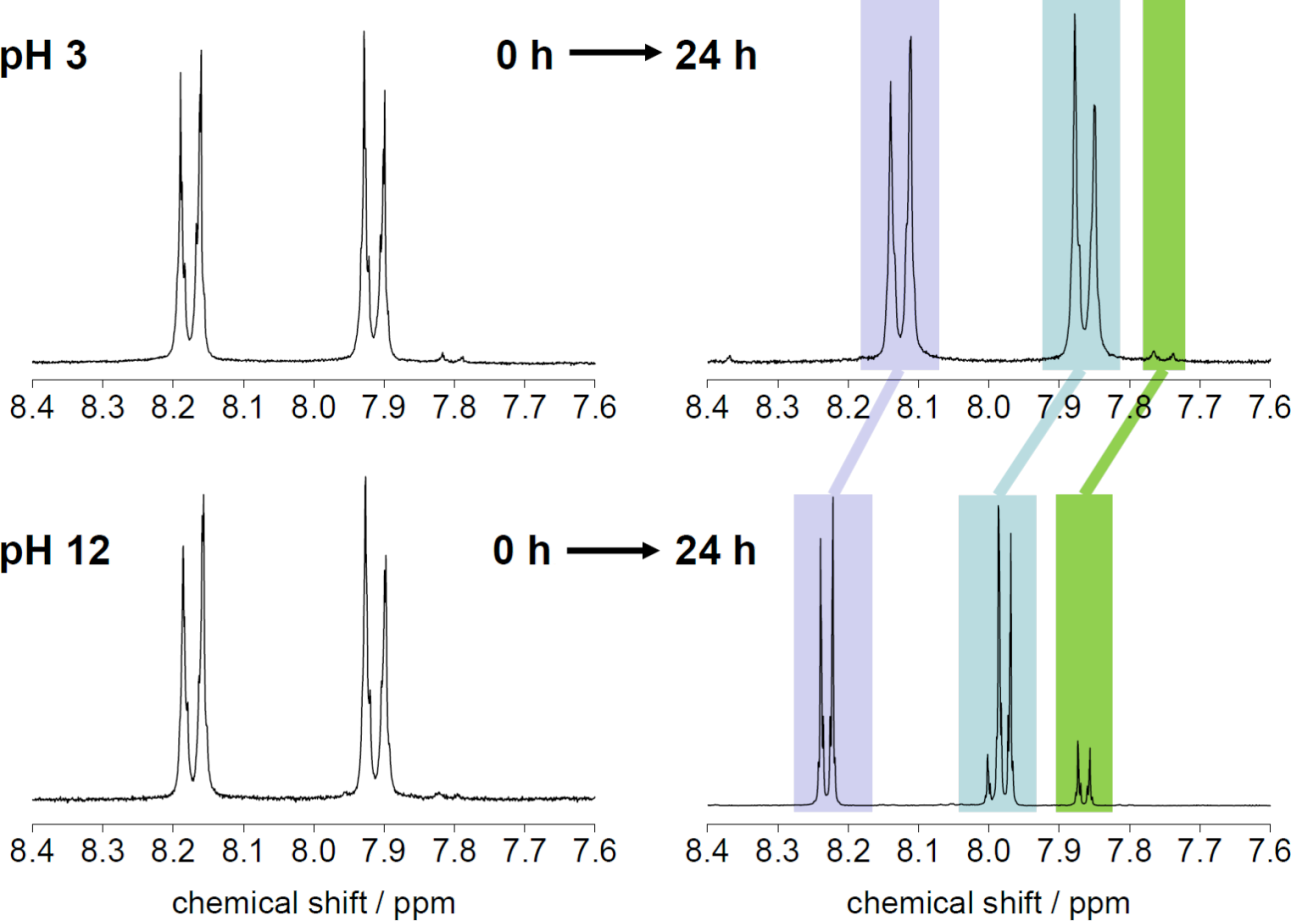
Representative NMR spectra of CBAmPEG after incubation at pH 3 for 0 h (left top) and 24 h (right top) and after incubation at pH 12 after 0 h (left bottom) and 24 h (right bottom). All data are for 37 °C. See Experimental section for signal assignments. Colored areas highlight the corresponding signals that are shifted after treatment at pH 12.

To determine the extent of ester cleavage in the pure polymer, CBAmPEG was incubated in D_2_O solutions of pH 3, 6, 9, and 12 at 37 °C for 24 h. After different time intervals, aliquots were taken and the samples were directly investigated using ^1^H NMR spectroscopy (Figure 3).

At pH 3, 6, and 9 no significant change of the polymer was observed and all NMR spectra indicate that CBAmPEG remains intact over the measurement period of 24 h. A small signal at 7.8 ppm indicates that ca. 1% of unreacted cyanobenzoic acid is still present in the samples and could not be removed.

At pH 12, however, changes occur: (i) the signal at ca. 7.8 ppm from the cyanobenzoic impurity becomes slightly more intense and (ii) a new signal is visible at 8.0 ppm. These data show that at pH 12 the polymer is indeed cleaved at the ester moiety. Interestingly, however, the efficiency of the cleavage is rather low and integration of the NMR signals shows that even at pH 12, only ca 12% of the polymer is cleaved.

Finally (iii), the spectra obtained from samples at pH 12 after 24 h are shifted compared to the signals observed for the samples held at pH 3 and the initial sample at pH 12. We currently speculate that this is due to some additional reaction of the nitrile group in the basic environment, but neither IR nor Raman spectroscopy show chemical changes in the –CN group. Overall, NMR spectroscopy shows that the polymer is very stable up to pH 9 and even at pH 12, the ester hydrolysis is only ca. 12 % in 24 h.

As the observation of the polymer degradation using NMR spectroscopy has proven difficult in the case of the polymers bound to the SNP surface, we have used zeta-potential measurements to evaluate the ester cleavage on the particles. Indeed, these data confirm the hydrolysis of the ester bond in CBAmPEG@SNPs incubated at pH 12 (Table 1). The zeta potentials are shifted towards more negative values (around −40 to −50 mV) for samples incubated in dispersions with a pH of 12. In contrast, the zeta potentials measured for samples treated at lower pH are around −20 to −30 mV.

Unlike the zeta potential, the hydrodynamic radius *R*_h_ (as determined by DLS) remains the same at all pH values and over the entire time of incubation, although there is a fairly large batch-to-batch variation. This also applies to samples treated at much longer reaction times up to 66 h. The fact that occasionally two populations with different hydrodynamic radii are observed is assigned to the presence of rather broad particle size distributions. DLS sometimes detects bimodal size distributions and in other cases monomodal but very broad distributions. This is also reflected in the rather large deviations from the mean value of *R*_h_ (Table 1) and is consistent with the UV–vis spectra (Figure 2).

## Discussion

Stable nanoparticle dispersions are currently of high interest [4–5]. Arguably, the most common stabilizer for gold and silver nanoparticles is PEG and the most common anchor group is the thiol/thiolate group. As stated in the introduction, there may however be situations where the thiol group may be unwanted. The new stabilizer presented here, CBAmPEG, is a viable alternative for at least some cases. The new polymer is easily accessible in good yields. NMR spectroscopy, IR spectroscopy, elemental analysis, and GPC show that the polymer is clean and has a monomodal (although somewhat broad, depending on the PEG used for the reaction) size distribution.

Moreover, the interaction of the cyano group with the silver surface is reasonably strong [36,38–43] and provides a good attachment of CBAmPEG to the NP surface. UV–vis spectroscopy suggests that a smaller fraction of the nanoparticles may aggregate upon modification with the polymer. There is, however, no indication of precipitation. This suggests that the polymer is a viable stabilizer for the SNPs. Finally, CBAmPEG is stable up to pH 9 before some degradation via cleavage of the ester bond is observed.

Consistent with the data on the pure CBAmPEG polymer, zeta-potential measurements show that no ester cleavage occurs up to pH 9, but we do observe a shift in the zeta potential towards more negative values after incubation of CBAmPEG@SNPs at pH 12. This is due to the fact that ca. 12% of the polymers attached to the SNP surface are cleaved at pH 12. This produces a negatively charged carboxylate (from 4-cyanobenzoic acid attached to the SNP surface) and a neutral alcohol (from the free PEG chains released from the surface). The –OH group does not affect the surface charge at pH 12 but the higher number of negative charges from the cleaved benzoic acid units on the SNP surface reduces the zeta potential. As the fraction of cleaved polymer is rather low, we never observed particle aggregation or sedimentation and the data thus show that the new polymer is a viable stabilizer for SNP over a wide pH range.

The fact that the particles already have a negative zeta potential at the beginning of the experiment is due to the presence of residual citrate and nitrate ions adsorbed on the particle surface that are not removed during CBAmPEG@SNP synthesis. This is confirmed by Raman spectroscopy, which detects carboxylate groups (from citrate) and NO_2_ vibrations from residual nitrate ions (from AgNO_3_), both of which contribute to a negative zeta potential even after polymer grafting but prior to polymer cleavage.

Finally, it is important to note that DLS does not detect a change in the hydrodynamic radius, *R*_h_, upon polymer degradation on the particle surface. In case of large amounts of cleaved polymer on the particle surface, aggregation and a concurrent increase of *R*_h_ may be expected. We currently assign the unchanged *R*_h_ to the fact that only a small fraction of the polymer chains is cleaved over the course of the reaction. As a result, the polymer shell on the particle surface is still intact and provides adequate steric stabilization. Possibly, the steric effect is further complemented by a contribution from electrostatic repulsion [55–56] as indicated by the zeta potential of the particles, which is shifted to more negative values after ester cleavage.

Overall the data thus demonstrate that CBAmPEG is an efficient stabilizer for silver nanoparticles. At low and neutral pH values it appears to stabilize the particles by a combination of steric repulsion (via the PEG coating) and a contribution from electrostatics arising from residual citrate and nitrate anions that still remain on the SNP surface even after washing. After treatment at high pH values and the corresponding cleavage of some of the stabilizer, the steric contribution is likely less pronounced (as some of the PEG is removed from the surface) and the contribution from electrostatic stabilization is likely somewhat stronger because the negatively charged carboxylate groups (from the cyanobenzoic acid moieties) also contribute to charging the SNP surface.

## Conclusion

The current article describes synthesis and performance of α-((4-cyanobenzoyl)oxy)-ω-methyl poly(ethylene glycol), the first polymeric stabilizer for metal nanoparticles based on a cyano rather than a thiol or thiolate anchor group. The polymer is quite stable at different pH conditions and is only cleaved at pH 12 or higher. Even after cleavage of the stabilizer attached to the SNP surface, however, the nanoparticle dispersions remain stable and no flocculation or precipitation is observed. As a result, the study shows that (i) cyanobenzoic acid-based polymeric stabilizers are applicable over a wide pH range and (ii) some stabilizer degradation may take place without causing a subsequent destabilization of the nanoparticle dispersion. The new polymeric stabilizer CBAmPEG may thus be interesting for application in biology, medicine, or diagnostics.
